# On the use of DNA as a linker in antibody-drug conjugates: synthesis, stability and *in vitro* potency

**DOI:** 10.1038/s41598-020-64518-y

**Published:** 2020-05-06

**Authors:** Igor Dovgan, Anthony Ehkirch, Victor Lehot, Isabelle Kuhn, Oleksandr Koniev, Sergii Kolodych, Alexandre Hentz, Manon Ripoll, Sylvain Ursuegui, Marc Nothisen, Sarah Cianférani, Alain Wagner

**Affiliations:** 1 Bio-Functional Chemistry (UMR 7199), LabEx Medalis, University of Strasbourg, 74 Route du Rhin, 67400 Illkirch-Graffenstaden, France; 2 BioOrganic Mass Spectrometry Laboratory (LSMBO), IPHC, University of Strasbourg, 25 rue Becquerel, 67087 Strasbourg, France; 3 Syndivia SAS, 650 Boulevard Gonthier d’Andernach, 67400 Illkirch-Graffenstaden, France; 4 IPHC, CNRS, UMR7178, University of Strasbourg, 67087 Strasbourg, France

**Keywords:** Chemical modification, DNA, Drug delivery, Proteins

## Abstract

Here we present the synthesis and evaluation of antibody-drug conjugates (ADCs), for which antibody and drug are non-covalently connected using complementary DNA linkers. These ADCs are composed of trastuzumab, an antibody targeting HER2 receptors overexpressed on breast cancer cells, and monomethyl auristatin E (MMAE) as a drug payload. In this new ADC format, trastuzumab conjugated to a 37-mer oligonucleotide (ON) was prepared and hybridized with its complementary ON modified at 5-end with MMAE (cON-MMAE) in order to obtain trastuzumab-DNA-MMAE. As an advantage, the cON-MMAE was completely soluble in water, which decreases overall hydrophobicity of toxic payload, an important characteristic of ADCs. The stability in the human plasma of these non-engineered ON-based linkers was investigated and showed a satisfactory half-life of 5.8 days for the trastuzumab-DNA format. Finally, we investigated the *in vitro* cytotoxicity profile of both the DNA-linked ADC and the ON-drug conjugates and compared them with classical covalently linked ADC. Interestingly, we found increased cytotoxicity for MMAE compared to cON-MMAE and an EC50 in the nanomolar range for trastuzumab-DNA-MMAE on HER2-positive cells. Although this proved to be less potent than classically linked ADC with picomolar range EC50, the difference in cytotoxicity between naked payload and conjugated payload was significant when an ON linker was used. We also observed an interesting increase in cytotoxicity of trastuzumab-DNA-MMAE on HER2-negative cells. This was attributed to enhanced non-specific interaction triggered by the DNA strand as it could be confirmed using ligand tracer assay.

## Introduction

Antibody-drug conjugates (ADCs) are promising therapeutic agents used mainly for cancer indications^1,2^. There are currently seven ADCs on the market, including three ADCs approved by the US Food and Drug Administration (FDA) in 2019^3^. Conjugation of highly potent cytotoxic drugs with antibodies recognizing the antigens overexpressed on cancer cells affords ADCs, which enable the delivery of the cytotoxic payload into the tumor cells in a controlled and selective manner. Following internalization and proteolytic cleavage, the drug induces tumor cell dysfunction and apoptosis. Classical approaches for the preparation of ADCs from native antibodies are based either on drug conjugation to exposed lysine residues or to cysteine residues generated by a reduction of interchain disulfide bonds^4^. Alternatively, protein engineering and enzymatic approaches have been actively used for production of homogeneous ADCs^5,6^. For more information about advancements in antibody, linker, and warhead technologies, we refer readers to several excellent reviews on these topics^7–9^.

**Figure 1:**
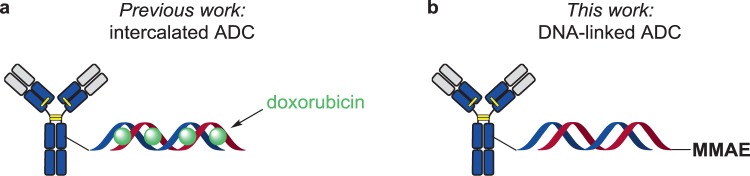
(**a**) Doxorubicin intercalated into antibody-conjugated DNA. (**b**) drug-conjugated DNA hybridized to to the complementary, antibody-conjugated DNA strand.

We have recently reviewed antibody-oligonucleotide conjugates (AOCs) for applications as therapeutic and detection agents. Interestingly, AOCs have been used for targeted therapy as carriers of doxorubicin drug which was intercalated between the CG base pairs of the AOC’s double-strand DNA (Fig. 1a)^10–12^. The Gothelf group has shown that a homogeneous anti-EGFR-dsDNA conjugate loaded with doxorubicin (~ 8 drugs per 21 bp DNA) had enhanced cytotoxicity to EGFR^+^ cells while being more tolerant to EFGR^-^ cells than free doxorubicin^12^. This opens a new avenue for development of targeted drug delivery systems based on AOCs, which had mainly been used as therapeutic agents in the context of small interfering RNA (siRNA) delivery^13^.

Here we describe another ADC format, in which a drug is covalently linked to an ON, allowing non-covalent linkage to the antibody via DNA base-pairing (Fig. 1b). This approach could be advantageous for the fast screening of new drug candidates by using a library of different Ab-ON and cON-drug conjugates. Another aspect of this study was devoted to the evaluation of the impact of the DNA on the ADC’s stability and potency *in vitro*. Furthermore, this format can be easily combined with intercalating drugs, allowing the preparation of dual-drug ADCs.

## Result and discussion

**Scheme 1:**
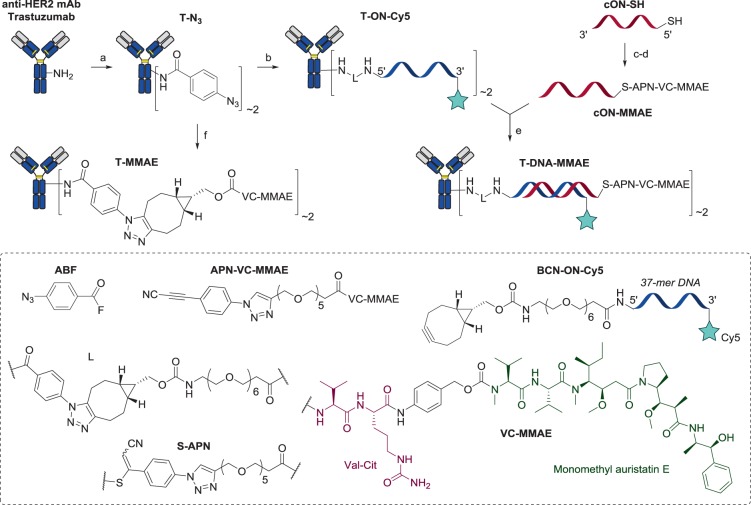
Preparation of the DNA-linked ADC. Reaction conditions: a) ABF (3 eq.) in PBS 1×, pH 7.5, 30 min, 25 °C; b) ON-BCN (2.9 eq.), 20 h, 25 °C; c) TCEP (100 eq.) in PBS 1×, pH 7.5, 2 h, 37 °C; d) APN-VC-MMAE (1.2 eq.) in 1/1 of DMSO/PBS 1×(pH 7.5, 5 mM EDTA), 16 h, 25 °C; e) in PBS 1×, pH 7.5, 20 min, 25 °C; f) BCN-VC-MMAE (4 eq.), 18 h, 25 °C.

To prepare DNA-linked ADCs in a versatile manner, we envisioned a parallel synthesis strategy, where both the Ab-ON and the cON-drug conjugates were prepared separately and then mixed to generate the desired ADC (Scheme 1).

We selected the anti-HER2 monoclonal antibody (mAb) trastuzumab, which is widely used in ADCs for breast cancer indications, including two FDA-approved ADCs: trastuzumab emtansine (Kadcyla)^14^ and trastuzumab deruxtecan (Enhertu)^15^. In order to attach ONs to the antibody, we exploited our previously described plug-and-play strategy^16,17^. Briefly, trastuzumab was reacted with 3 equiv. of 4-azidobenzoyl fluoride to obtain antibody-azide conjugates **T-N**_**3**_ with an average degree of conjugation (DoC) of 2.1^16^. This conjugate was then subjected to strain-promoted alkyne-azide cycloaddition (SPAAC) with ON-bicyclononyne (BCN-ON) derivative of 37-mer ON **BCN-ON-Cy5** (see SI for the synthesis) to afford AOC **T-ON-Cy5**. The design of the 37-mer ON was based on the several requirements: 1) the length of 20–40 nucleotides for sufficient hybridization strength; 2) minimal secondary structure of ONs to ease their hybridization at room temperature; 3) DNA’s melting point high enough to limit dehybridization *in vivo* (security margin of 30 °C was applied to get 37-mer ON with Tm of 66.4 °C).

**Figure 2:**
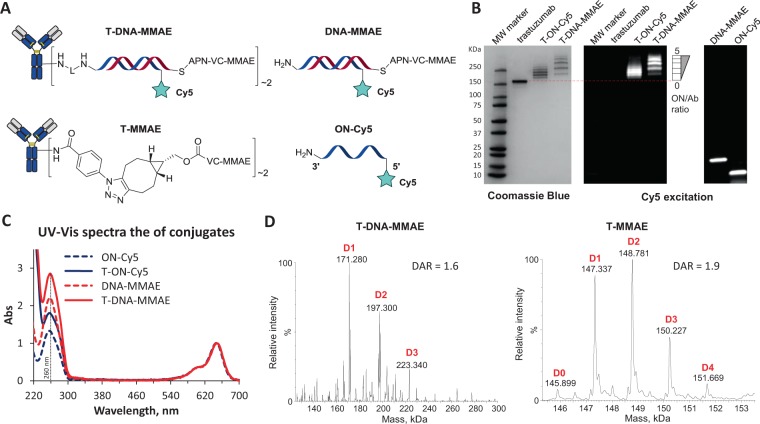
(**A**) Structures of ADC **T-MMAE** and **T-DNA-MMAE**. (**B**) Denaturing SDS PAGE (4–15%) analysis showed complete hybridization of **T-DNA-MMAE** and no sign of unconjugated payloads (bands of **DNA-MMAE** and **ON-Cy5** served as a positive control; the cropped parts of the same gel are displayed for the fluorescent gel, for the full-length gel see SI Figure S12). (**C**) Normalized UV-Vis spectra of the conjugates demonstrated successful conjugation of about two payloads per trastuzumab (average DoC_UV_ = 2.1, see SI Table S1). (**D**) Deconvoluted native MS of the ADCs.

At the same time we conjugated the drug to the complementary ON (cON). As a drug we used monomethyl auristatin E, functionalized with a cleavable valine-citrulline linker (VC-MMAE) and a *p*-aminobenzyloxycarbonyl group. This drug-linker format is used in three FDA-approved ADCs: brentuximab vedotin (Adcetris), polatuzumab vedotin (Polivy) and enfortumab vedotin (Padcev); all having an average drug-to-antibody ratio (DAR) of ~ 4^3,18^. For drug conjugation to the ON, we applied a thiol-conjugation technology previously developed in our group, which is based on selective and irreversible thiol tagging with 3-arylpropiolonitriles (APN)^19–22^. Thus, a thiol-modified 37-mer ON was conjugated to APN-VC-MMAE to afford a conjugate **cON-MMAE** which was purified by HPLC in good yield. Remarkably, this drug payload was completely soluble in water at micromolar range and was at least 138 times more soluble than **APN-VC-MMAE** (see SI, **APN-VC-MMAE** synthesis), thus demonstrating the applicability of DNA linker to increase the solubility of hydrophobic drugs. **cON-MMAE** was used to anneal with AOC **T-ON-Cy5** to yield ADC **T-DNA-MMAE**, which was then purified by gel filtration chromatography to remove excess of DNA payload. To compare this ADC to classical covalently linked ADC, **T-MMAE** was prepared with an average DAR of 1.9 by reacting **T-N**_**3**_ with **BCN-VC-MMAE** (Fig. 2A). Moreover, as MMAE-based ADCs typically use cysteine residues instead of lysines for drug-linker conjugation to antibody, we have prepared a Cys-linked ADC **T-Cys-MMAE** with an average DAR of 4.0 by reacting partially reduced trastuzumab with **APN-VC-MMAE** (see SI Figure S9).

SDS PAGE analysis of **T-DNA-MMAE** showed multiple bands, each of which corresponded to different DNA/Ab ratio ranging from 1 to 5 and the average DoC of 2–3. This is in accordance with the average DoC value of **T-N**_**3**_, demonstrating completeness of the SPAAC reaction. No sign of unconjugated DNA payload was detected (Fig. 2B). UV-Vis spectra of the **T-DNA-MMAE** and **T-ON-Cy5** conjugates were recorded and normalized to their absorption value at 650 nm (Fig. 2C). Absorption at 260 nm was higher for **T-DNA-MMAE** than for **T-ON-Cy5**, confirming the presence of higher amount of DNA in this conjugate. Using absorption value at 650 nm and the amount of antibody found by BCA assay, we estimated the average DoC value of 2.1 for both conjugates (see SI Table S1).

Finally, we performed native MS analysis experiments for intact mass measurement and DAR determination of DNA-linked ADC. The manual desalting step prior to native MS experiment was not possible for **T-DNA-MMAE** due to precipitation in ammonium acetate buffer (150 mM, pH 7.4). An alternative was to analyze the ADC by native SEC-MS, providing an online desalting step. Deconvoluted mass spectrum of **T-DNA-MMAE** (Fig. 2D) showed an average DAR of 1.6 with drug distribution ranging from 1 to 3. Interestingly, higher DAR species observed in SDS PAGE analysis were not detected by mass spectrometry. It was previously noticed that the ON’s charge can impact the ionization efficiency, especially for higher DAR species and does not fully reflect the entire sample composition^23^.

Stability in human plasma is an important characteristic of therapeutic bioconjugates. In order to protect DNA from nuclease cleavage, xeno nucleic acids, such as L-configured ONs^24^ or peptide nucleic acid^25–27^ are typically used. The nuclease cleavage of DNA is an important factor to take into account during developing DNA-linked ADC. Unfortunately, little is known about protein-conjugated DNA stability in human plasma from the literature.

**Figure 3:**
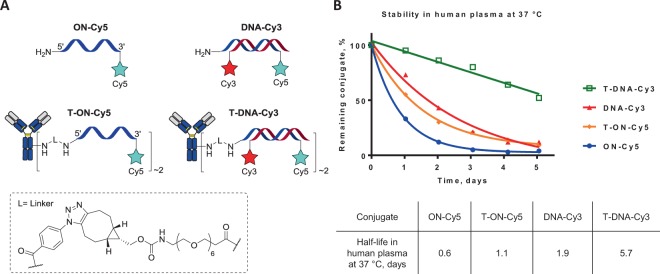
(**A**) Structure of the conjugates. (**B**) Stability of the conjugates incubated in human plasma at 37 °C, determined from in-gel fluorescence. The half-life values were found from one phase decay fitting of data plot, except for T-DNA-Cy3 the linear plot was used.

We therefore tested the stability of conjugates having either single or double-stranded ON in human plasma. (Fig. 3). The conjugates **DNA-Cy3** and **T-DNA-Cy3** were obtained by hybridization of **ON-Cy5** and **T-ON-Cy5** with complementary 37-mer **cON-Cy3**, respectively. These conjugates had both Cy5 and Cy3 fluorophores at the 3-end of ON, which allowed the conjugate detection and quantification over time by in-gel fluorescence.

For the stability test, the conjugates were incubated in human plasma at 37 °C. Aliquots of each probe were taken every day, diluted with water and frozen at −20 °C. The resulting probes were then analyzed by SDS PAGE and in-gel fluorescence was measured (see SI Figure S10). Cleavage of the ON linker by nucleases should be accompanied by a decrease in fluorescence of the bands corresponding to the conjugate.

It was found that **ON-Cy5** gradually disappeared over incubation time and had a half-life of 0.6 days in human plasma (Fig. 3B).

Interestingly, the half-life of **T-ON-Cy5** (t_1/2_ of 1.1 days) was twice higher than that of the unconjugated **ON-Cy5**. This could be explained by a steric factor of the antibody, which shields the ONs from nuclease cleavage^28,29^. Even higher stability was observed for the double-stranded format **DNA-Cy3** (t_1/2_ of 1.9 days) and **T-DNA-Cy3** (t_1/2_ of 5.7 days), which displayed half-lives 3- and 10-times higher than that of **ON-Cy5**. This suggests that this DNA-linked ADC could be a suitable candidate for *in vivo* use even without resorting to DNA engineering. Namely, these results showed that the stability of this non-engineered AOCs is close to that of maleimide-based antibody conjugates (38% degradation after 5 days)^30^.

In-gel fluorescence showed preservation of the sharp lines corresponding to **T-DNA-Cy3** over time (see SI Figure S10). The specific mass loss of the payload and appearance of the distinct band corresponding to **DNA-Cy3** would suggest that the nuclease cleavage site is located near the 5’-terminus of **ON-Cy5**.

**Figure 4:**
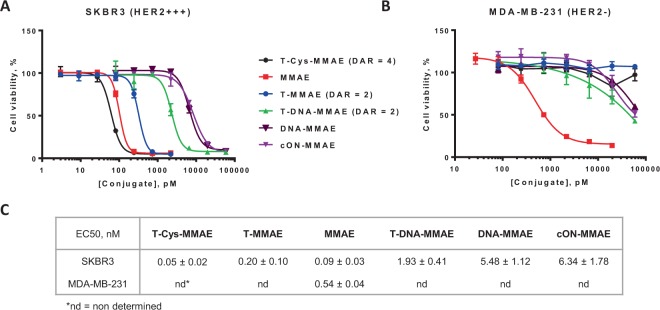
*In vitro* cytotoxicity on (**A**) SKBR3 and (**B**) MDA-MB-231 cell lines. (**C**) EC50 values of the conjugates determined using four-parameter logistic fitting. EC50 ± SD values from three independent experiments.

We then embarked on the *in vitro* study by performing cytotoxicity MTT assays of the various ADC constructs on SKBR3 (HER2-positive) and on MDA-MB-231 (HER2-negative) cell lines (Fig. 4).

We first noticed that the cytotoxicity of **cON-MMAE** and **DNA-MMAE** on the SKBR3 cells was similar and the conjugates had a quite high median effective concentration (EC50 = 6.34 ± 1.78 and 5.48 ± 1.12 nM, respectively) compared to that of the unmodified **MMAE** (EC50 = 0.09 ± 0.03 nM). We attributed this interesting result to a lower cell penetration induced by the addition to the drug of a large number of negative charges carried by the ONs.

Similarly we observed that **T-DNA-MMAE (**EC50 = 1.93 ± 0.41 nM) was less effective than **T-MMAE** (EC50 = 0.20 ± 0.10 nM) or **T-Cys-MMAE** (EC50 = 0.05 ± 0.02 nM). Here again, the addition of negative charges could account for this weaker cytotoxicity.

Interestingly, despite this apparent drawback, if one compares the relative cytotoxicity of **MMAE**/**T-MMAE** and **DNA-MMAE**/**T-DNA-MMAE** one can notice that in the first case the drug is more potent that the conjugate, while, in the second case, the conjugate is more potent than the drug. Even though it seems too early to draw definitive assertion, these results could suggest a way to design ADC for which premature deconjugation would lead to a less toxic drug and possibly afford a strategy to reduce potential side effects resulting from drug deconjugation.

A second interesting effect came from studying the cytotoxicity of our construct on the HER2 negative MDA-MB-231 cell line. **MMAE** and **T-MMAE** (or **T-Cys-MMAE**) behaved as expected: the drug being highly toxic and the conjugate showing no toxicity. In the case of DNA-based conjugates we surprisingly observed that **cON-MMAE**, **DNA-MMAE** behaved similarly to antibody conjugated **T-DNA-MMAE**. The “protecting” effect toward non-HER2 expressing cells brought by conjugation of the drug to the antibody was somehow reduced at high concentrations. Interestingly, the doxorubicin intercalated EGFR-dsDNA has also been reported to be toxic for antigen-negative cells at high concentrations, which was assumed to be due to ADC instability over 48 h of incubation in cell medium^12^.

We attributed this effect to nonspecific interaction of ONs with the cell surface. In order to dig deeper into this assumption, we engaged LigandTracer assay in live cells to evaluate the binding kinetics difference between mAb and AOC.

**Figure 5:**
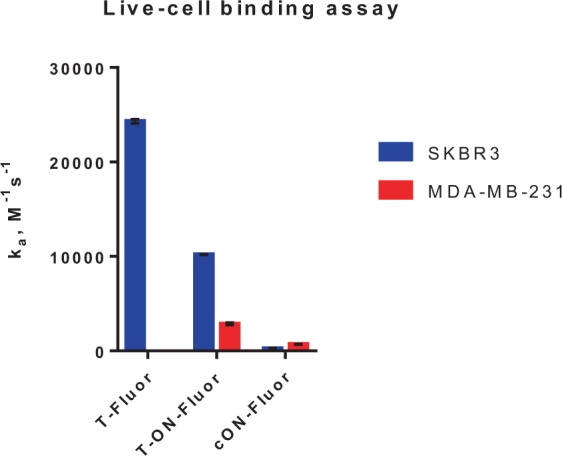
Comparison of association constant k_a_ for fluorescein-labeled antibody, AOC and ON.

To this end, trastuzumab and 37-mer ON-conjugated trastuzumab were labeled with fluorescein isothiocyanate to afford assay-traceable conjugates, **T-Fluor** and **T-ON-Fluor**, respectively. Complementary 37-mer ON labeled with fluorescein, **cON-Fluor**, was used as ON’s binding control. The SKBR3 and MDA-MB-231 cells were exposed to the fluorescein-labeled conjugates and the fluorescence intensity of cells was monitored over time. The increase of fluorescence signal was corrected from a background value of the plastic support (Figure S11) and was used to calculate the association constant k_a_ shown on Fig. 5.

First, the SKBR3 binding was in line with cytotoxicity assays showing significantly lower association constant for AOC (1.02 · 10^4^ M^−1^s^−1^) compared to mAb (2.43·10^4^ M^−1^s^−1^). Moreover, on the HER2-negative MDA-MB-231 cells, **T-Fluor** behaves as expected with no detectable interactions, while **T-ON-Fluor** showed some nonspecific binding with a k_a_ value of 0.3 · 10^4^ M^−1^s^−1^. Although much weaker than the effect induced by HER2 targeting, this nonspecific binding correlates with the cytotoxicity of DNA-linked ADC measured on MDA-MB-231 cells at sub-micromolar range. Interestingly, the low association of **cON-Fluor** with both cell lines was also detected (~ 300–700 M^−1^s^−1^) and was in accordance with previously reported results of AOCs and ONs association with cell membranes^31,32^.

## Conclusion

In summary, we prepared a DNA-linked ADC by hybridization of an antibody-ON conjugate with a cON-drug conjugate. The structure of this non-covalent ADC was confirmed by SDS PAGE and MS analysis, as well as by UV-Vis spectrophotometry. The *in vitro* test of stability in human plasma demonstrated surprisingly good stability of double-stranded DNA-antibody conjugate, which proved to be close to that of a maleimide-based ADC.

*In vitro* cytotoxicity assay of DNA-linked ADC **T-DNA-MMAE** showed successful targeting of DNA-drug payload to HER2-positive cell line and induced cell death at nanomolar concentrations (EC50 = 1.93 ± 0.41 nM). This EC50 value is higher than that of covalently linked ADC **T-MMAE** (EC50 = 0.20 ± 0.10 nM) or **T-Cys-MMAE** (EC50 = 0.05 ± 0.02 nM), probably because of the negative charges of DNA that may affect internalization/binding or drug processing.

From the point of view of possible therapeutic use, the effect of a DNA linker on the behavior of the conjugate appears somehow contradictory. On one hand, it reduces the toxicity of the free drug by reducing its cell penetration, which is positive in case of premature deconjugation in the bloodstream. One the other hand, it potentially increases the off-target toxicity on low antigen-expressing cells, presumably due to nonspecific interaction of the nucleic acid-based linker with the cell surface.

These latest observations might be of high interest, especially for the AOC design in the context of siRNA, or microRNA delivery. Further studies will be conducted to investigate the generality of this phenomenon.

## Methods

### General experimental procedures

Protein concentration of antibody stock solution (PBS 1/20×, pH 7.5) was determined by UV absorbance using a NanoDrop spectrophotometer (Thermo Fisher Scientific, Illkirch, France). The concentration of antibody conjugates was measured using the BCA Protein Assay Kit (Ref. 23225, Thermo Fisher Scientific). The antibody conjugates were purified by gel filtration chromatography on Superdex 200 Increase 10/300 GL (GE Healthcare).

### Materials

All reagents were obtained from commercial sources and used without prior purifications. Dry solvents were obtained from Sigma-Aldrich. The 37-mer ONs used in this study were obtained from Integrated DNA Technologies:

**Table:** 

Name	5-end	3-end	Sequence (5’→3’)
**ON-Cy5**	AmMC12	Cy5Sp	AAGATACGAATTCGGGTGTTCTGCTGGTAGTGGTCGG
**NH2-ON**	AmMC12	-	
**cON-Cy3**	AmMC12	Cy3Sp	CCGACCACTACCAGCAGAACACCCGAATTCGTATCTT
**cON-Fluor**	FAM	-	
**cON-SH**	ThioMC6D	-	

### HPLC purification of ONs

The purifications of modified ONs were carried out on a Shimadzu system consisting of two LC 20-AD pumps, an SPD 20-A detector, a SIL 20-A autosampler, and a SunFire C18 column (4.6 × 150 mm i.d., 5 µm, Waters). HPLC parameters were as follows: flow rate of 1 mL/min, mobile phase A: 50 mM TEAA in water, mobile phase B: 50 mM TEAA in acetonitrile. The detection was done at 260 nm. Gradient: from 15% to 35% of mobile phase B in 30 min (for BCN-ON-Cy5) and from 10% to 40% of mobile phase B in 30 min (for cON-MMAE). After HPLC the conjugates were lyophilized using a SpeedVac (Savant, Illkirch, France).

### SDS PAGE analysis

Non-reducing glycine-SDS-PAGE was performed on 4–15% Mini-PROTEAN TGX Gel (Ref. 4561084, Bio-Rad)^30^. To samples containing antibody conjugates (0.1 mg/mL, 24 μL) was added 8 μL of 4x non-reducing Laemmli SDS sample buffer (Ref. J63615, Alfa Aesar) and heated at 95 °C for 3 minutes. The samples (10 µL) were loaded into wells and the gel was run at constant voltage (200 V) for 35 min using TRIS 0.25 M - Glycine 1.92 M - SDS 1% as a running buffer. Fluorescence was visualized on the ImageQuant LAS 4000 series (GE Healthcare Life Sciences) prior to staining with Coomassie Blue.

### Samples preparation for native MS analysis

Antibody conjugates (2 mg/mL, 50 µL, 100 µg) in PBS (1/20×, pH 7.4) were deglycosylated by incubating (37 °C, 2 h) with Remove-iT Endo S (0.5 µL, 200 U/µL, New England BioLabs) and 10X GlycoBuffer 1 (5 µL, New England BioLabs). The deglycosylated antibody conjugates were desalted against 150 mM ammonium acetate solution buffered at pH 7.4 using six cycles of concentration/dilution on micro-concentrators (Vivaspin, 30 kD cutoff, Sartorius, Gottingen, Germany). Protein concentration was determined by UV absorbance using a NanoDrop spectrophotometer (Thermo Fisher Scientific, Illkirch, France).

### Native MS analysis

Native MS analyses were performed in positive mode, on an ESI-TOF (LCT, Micromass, Altrincham, UK) upgraded by MS Vision (MS Vision, Almere, Netherlands) coupled with an automated chip-based nanoESI infusion source (Triversa Nanomate, Advion Ithaca, USA)^33^. Instrumental parameters were tuned to ensure the transmission of high molecular weight species and preservation of potential non-covalent interactions disruption. The acceleration voltage was set to 120 V and the pressure in the interface region of the mass spectrometer was 6.0 mbar. Acquisitions were performed during 2 min with a scan time of 4 s after external calibration performed with a 2 mg/mL solution of cesium iodide in 2-propanol/water (50/50 v/v). MS data interpretations were performed using Mass Lynx V4.1 (Waters, Manchester, UK).

### Samples preparation for native SEC-MS analysis

AOCs (100 µg) in PBS 1x buffer were deglycosylated by incubating (37 °C, 16 h) with Remove-iT Endo S (1 µL, 200 U/µL, New England BioLabs) prior to the native SEC-MS analysis.

### Native SEC-MS analysis

An Acquity UPLC H-class system (Waters, Manchester, UK) hyphenated to a Synapt G2 HDMS mass spectrometer (Waters, Manchester, UK) was used for the online SEC-native MS instrumentation. The SEC column was an an AdvanceBio SEC (50 mm × 4.6 mm, 2.7 µm, 300 Å) from Agilent Technologies (Wilmington, DE, USA). The elution was carried out in isocratic mode with an aqueous mobile phase composed of 100 mM ammonium acetate pH 6.8 at a flow-rate of 100 µL/min.

The Synapt G2 HDMS was operated in the positive mode with a capillary voltage of 3.0 kV. The sample cone and pressure in the interface region were set to 180 V and 6 mbar, respectively^34,35^. Acquisitions were performed in the m/z range 1000–8000 for fast desalting experiments and in the m/z range 1000–10000 with a 1.5 s scan time. External calibration was performed using singly charged ions produced by a 2 g/L solution of cesium iodide in 2-propanol/water (50/50 v/v). MS data interpretations were performed using Mass Lynx V4.1 (Waters, Manchester, UK).

### DoC calculation from MS analysis

The average DoC values from native MS were calculated by using Eq. 1. These results were derived from the relative peak intensities in deconvoluted mass spectra.

1 $$DoC=\frac{\sum n\cdot {I}_{n}}{\sum {I}_{n}}$$

where *I*_*n*_ is relative peak intensity of conjugates with *n* add-on molecules per antibody.

### DoC calculation from UV-Vis spectroscopy

The average DoC values were calculated as a ratio between the dye concentration measured by NanoDrop spectrophotometer and antibody concentration obtained from BCA assay. Next extinction coefficients were applied for the calculation of dye concentration: 73 000 M^−1^cm^−1^ at 495 nm for fluorescein and 250 000 M^−1^cm^−1^ at 650 nm for Cy5.

### Stability in human plasma

Human plasma was supplied by Etablissement Français du Sang (EFS, Strasbourg). The probes were tested at the same amount of ON per sample: conjugates **ON-Cy5** and **DNA-Cy3** (10.1 µM, 25 µL) and conjugates **T-ON-Cy5** and **T-DNA-Cy3** (0.5 mg/mL, 25 µL). The probes (25 µL) were mixed with human plasma (25 µL), filled with nitrogen and incubated at 37 °C. The aliquots (2 µL) were taken every day at certain time points, diluted with water (48 µL), frozen in liquid nitrogen and stored at −20 °C. The resulting samples (24 µL) were then subjected to SDS PAGE analysis and in-gel fluorescence scanning (see SI Figure S10). The remaining conjugate amount at each time point was calculated by dividing the fluorescence intensity of the conjugate at this point to its fluorescence intensity at the starting point.

### Cell cultures

Human breast adenocarcinoma cells SKBR3 (HER2-positive, ATCC HTB-30) and MDA-MB-231 (HER2-negative, ATCC HTB-26) were grown in cell medium: Dulbecco’s Modified Eagle’s Medium (DMEM) containing 4.5 g/L glucose, 2 mM L-Glutamine, supplemented with 10% fetal bovine serum (FBS), penicillin (100 units/mL), and streptomycin (100 μg/mL). Cells were maintained in a 5% CO_2_ humidified atmosphere at 37 °C.

### *In vitro* cytotoxicity assay

The day before experiment, *S*KBR3 and MDA-MB-231 cell lines were seeded in 96-well plates at 6000 cells/well in 100 μL fresh cell medium. The day of experiment, the medium was removed carefully and cells were incubated with conjugates (3–59049 pM in fresh cell medium, 100 µL/well, in triplicate) for 96 h. The MTT reagent (Sigma-Aldrich, 100 μL, 1 mg/mL in cell media) was added into each well and cells were incubated for 1.5 h at 37 °C. The medium was removed carefully and the blue precipitate was solubilized by adding 100 μL of DMSO. Cell viability was measured by quantifying absorbance at 570 nm using a 96-well plate reader (Flx-Xenius XM, Safas, Monaco). EC50 values were determined using four-parameter logistic fitting in GraphPad Prism 7.0. EC50 ± SDs were calculated from three independent experiments.

### Binding kinetics assay

The day before experiment, cells were seeded as 600 μL droplets with 10^6^ cells/mL near the edge of a circular cell dish (87 mm, Greiner, Germany) and were incubated at 37 °C overnight to allow their attachment to the dish surface. Two spots on the dish were used for SKBR3 cells, one was used for MDA-MB-231 cells and one spot was not treated (background reference area).

Prior to kinetic measurements, the cell medium was replaced with 3 ml fresh cell medium. The dish was placed in LigandTracer Green (Ridgeview Instruments)^36^. A baseline signal was collected for 30 min, and then a fluorescein-labeled conjugate was added in two increasing concentrations (9 and 27 nM) to record association. Dissociation of the conjugate was recorded after replacing the incubation solution with 3 ml fresh medium. Signals from cell and reference areas are recorded during every rotation, resulting in a background-substracted binding curve. Each concentration was incubated until sufficient curvature was obtained for subsequent extraction of kinetic parameters. Binding traces were analyzed with the evaluation software TraceDrawer 1.8.1 (http://www.ridgeview.eu/software/tracedrawer, Ridgeview Instruments) to determine k_a_, k_d_ and K_D_ according to the « one-to-one » model or Langmuir binding model.

## Supplementary information

Supplementary Information.
