# Supplementary material for: On the use of DNA as a linker in antibody-drug conjugates: synthesis, stability and in vitro potency
Source: Sci Rep. 2020 May 6;10:7691. doi: 10.1038/s41598-020-64518-y (PMC7203131; doi:10.1038/s41598-020-64518-y)
Supplement: Supplementary file 1 — Supplementary Information. [file 41598_2020_64518_MOESM1_ESM.docx]

**Supporting Information**

**On the use of DNA as a linker in antibody-drug conjugates: synthesis, stability and *in vitro* potency**

Igor Dovgan, Anthony Ehkirch, Victor Lehot, Isabelle Kuhn, Oleksandr Koniev, Sergii Kolodych, Alexandre Hentz, Manon Ripoll, Sylvain Ursuegui, Marc Nothisen, Sarah Cianférani and Alain Wagner^*^

Bio-Functional Chemistry (UMR 7199)

# LaBex MEDALIS, UMR 7199 F

# Faculté de Pharmacie

# Université de Strasbourg

# 74 route du Rhin

# CS 60024

# 67401 ILLKIRCH CEDEX

# Email: alwag@unistra.fr

# Compounds synthesis

### 4-((S)-2-((S)-2-((((bicyclo[6.1.0]non-4-yn-9-yl)methoxy)carbonyl)amino)-3-methylbutanamido)-5-ureidopentanamido)benzyl ((S)-1-(((S)-1-(((3R,4S,5S)-1-((S)-2-((1R,2R)-3-(((1S,2R)-1-hydroxy-1-phenylpropan-2-yl)amino)-1-methoxy-2-methyl-3-oxopropyl)pyrrolidin-1-yl)-3-methoxy-5-methyl-1-oxoheptan-4-yl)(methyl)amino)-3-methyl-1-oxobutan-2-yl)amino)-3-methyl-1-oxobutan-2-yl)(methyl)carbamate (**BCN-MMAE**)

Compound **1** was prepared as previously described.^1^ **NH_2_-VC-MMAE** was purchased from ACES Pharma (Princeton, NJ).

To the solution of **NH_2_-VC-MMAE** (1 eq., 0.1 M in DMSO, 50 μL, 5 μmol) was added **1** (2 eq., 0.1 M in DMSO, 100 μL, 10 μmol) and a mixture of DIPEA (10 eq., 0.5 M in pyridine, 100 μL, 50 μmol) and HOBt (4 eq., 0.2 M in pyridine, 100 μL, 20 μmol). The mixture was flushed with argon and incubated at 25 °C for 36 h. The crude reaction mixture was purified by preparative HPLC (detection at 254 nm; buffer A: H_2_O (0.05% of TFA); buffer B: MeCN; gradient from 5% to 95% B in 40 min) and promptly lyophilized to yield **BCN-MMAE** as a white solid. The solid was dissolved in DMSO-d6 (1 mL) and the concentration was determined by NMR (ERETIC method). Finally, **BCN-MMAE** (888 µl, 0.7 mM in DMSO-d6, 0.622 µmol) was obtained in 12% yield (Figure S1).

MS (ESI) *m/z*: 650.69 [M+2H]^2+^.

Figure S1. LC-MS analysis of **BCN-MMAE**

### tert-butyl 1-(1-(4-(cyanoethynyl)phenyl)-1H-1,2,3-triazol-4-yl)-2,5,8,11,14-pentaoxaheptadecan-17-oate (**4**)

*tert*-Butyl 4,7,10,13,16-pentaoxanonadec-18-ynoate **2** was synthesized as previously described.^2^ 3-(4-Azidophenyl)prop-2-ynenitrile **3** was purchased from Sigma Aldrich.

Compounds **3** (1 eq., 1.12 g, 6.66 mmol) and **2** (1 eq., 2.40 g, 1 mL, 6.66 mmol) were dissolved in THF (25 mL) and water (5 mL) was added to the mixture. To the obtained solution were added CuSO_4_ (0.10 g, 0.627 mmol) in water (0.5 mL) and sodium ascorbate (0.40 g, 2.02 mmol) in water (1 mL). The reaction mixture stirred for 5 minutes and another portion of CuSO_4_ (0.10 g, 0.627 mmol) and sodium ascorbate (0.40 g, 2.02 mmol) were added to the reaction mixture. To the resulting solution was added an equal volume of a saturated solution of NH_4_Cl, the organic fraction was separated, and the aqueous phase was extracted three times with EtOAc. Combined organic layers were dried over MgSO_4_, evaporated, and the crude product was purified by flash chromatography (Cyclohexane/EtOAc: 100/0 to 0/100 gradient in 20 min) to give compound **4** (2.11 g, 4 mmol, 60 %) as a yellowish oil.

**^1^H NMR (400MHz, CDCl_3_, δ ppm):** 8.14 (s, 1H), 7.88 (d, *J* = 8.3 Hz, 2H), 7.79 (d, *J* = 8.3 Hz, 2H), 4.79 (s, 2H), 3.81 - 3.55 (m, 18H), 2.49 (t, *J* = 6.5 Hz, 2H), 1.44 (s, 9H).

**MS (ESI) *m/z*:** 529.12 [M+H]^+^.

### 1-(1-(4-(cyanoethynyl)phenyl)-1H-1,2,3-triazol-4-yl)-2,5,8,11,14-pentaoxaheptadecan-17-oic acid (**5**)

To a solution of **4** (1 eq., 1.77 g, 3.35 mmol) in MeCN (50 mL) was added TFA (20 eq., 7.64 g, 4.97 mL, 67 mmol). The resulting reaction mixture was incubated for 5 days at room temperature. The solvent was evaporated and 100 mL of EtOAc were added. The organic layer was washed with water (3x 50 mL), brine (1x 50 mL), dried over MgSO_4_ and evaporated to yield the compound **5** (1.25 g, 2.65 mmol, 79 %) as a yellowish oil (Figure S2).

**^1^H NMR (400MHz, CDCl_3_, δ ppm):**8.29 (s, 1 H), 7.91 (d, *J* = 8.0 Hz, 2 H), 7.79 (d, *J* = 8.0 Hz, 2 H), 4.81 (s, 2 H), 3.83 - 3.53 (m, 18 H), 2.58 (t, *J* = 5.6 Hz, 2 H).

**MS (ESI) *m/z*:** 473.26 [M+H]^+^, 495.30 [M+Na]^+^.

Figure S2. LC-MS analysis of **5**

### 4-((19S,22S)-1-(1-(4-(cyanoethynyl)phenyl)-1H-1,2,3-triazol-4-yl)-19-isopropyl-17,20-dioxo-22-(3-ureidopropyl)-2,5,8,11,14-pentaoxa-18,21-diazatricosan-23-amido)benzyl ((S)-1-(((S)-1-(((3R,4S,5S)-1-((S)-2-((1R,2R)-3-(((1S,2R)-1-hydroxy-1-phenylpropan-2-yl)amino)-1-methoxy-2-methyl-3-oxopropyl)pyrrolidin-1-yl)-3-methoxy-5-methyl-1-oxoheptan-4-yl)(methyl)amino)-3-methyl-1-oxobutan-2-yl)amino)-3-methyl-1-oxobutan-2-yl)(methyl)carbamate (**APN-VC-MMAE**)

To the solution of **5** (1.2 eq., 35 mg, 74.1 μmol) in dry DMF (0.7 mL) was added 2-bromo-1-ethyl-pyridinium tetrafluoroborate (BEP, 1.2 eq., 20.3 mg, 74.1 μmol). The resulting mixture was incubated at 21 °C for 5 min and then **NH_2_-VC-MMAE** (1 eq., 69.3 mg, 61.7 μmol) and DIPEA (5 eq., 39.9 mg, 53.8 μL, 308 μmol) were added. The resulting mixture was stirred at 21 °C for 30 min and the crude product was purified by preparative HPLC to yield compound **APN-VC-MMAE** (49.7 mg, 31.5 μmol, 51 %) as a white solid (Figure S3 A). The solubility of **APN-VC-MMAE** in water was measured using UV-Vis spectrophotometry. Briefly, APN-VC-MMAE (1.2 µL, 16.6 mM, 20 nmol) was added in mQ water (98.8 µL) and the formed precipitate was mixed for 1 h at 25 °C and then centrifuged. The absorption of supernatant was measured using NanoDrop spectrophotometer (average A = 0.042) and the concentration was calculated using a calibration curve of **APN-VC-MMAE** in DMSO (Figure S3 B). The concentration of **APN-VC-MMAE** in water was about 2 µM, which corresponds to 1% of the starting drug (0.2 nmol/ 20 nmol) and at least 138 times less soluble than **cON-MMAE** (277 µM was obtained by dissolving of the lyophilized product in water).

**MS (ESI) *m/z*:** 789.70 [M+2H]^2+^.

Figure S3. (A) LC-MS analysis of **APN-VC-MMAE**. (B) Calibration curve of **APN-VC-MMAE** in DMSO obtained from UV-Vis spectrophotometry.

# Bioconjugation

## ****Preparation of ON conjugates****

***Precipitation with acetone***. ON solution (10-50 nmol) was diluted with water to the final volume of 300 µL and ON was precipitated by the addition of acetone (900 µL) and LiClO_4_ (20 µL, 3M in water). The sample was centrifuged at 15000 g for 8 min and a supernatant was discarded. The precipitate was dissolved in 300 µL of water and the procedure was repeated one more time.

### **Preparation of DNA-Cy3**

Conjugate DNA-Cy3 was prepared by mixing the ON-Cy5 and cON-Cy3 in an equimolar ratio in water.

### **Preparation of DNA-MMAE**

Conjugate DNA-MMAE was prepared by mixing the ON-Cy5 and cON-MMAE in an equimolar ratio in water.

### **Preparation of BCN-ON-Cy5**

**BCN-PEG6-PFP was synthesized as previously described.^3^ In a 2 mL Eppendorf tube, amino-modified oligonucleotide ON-Cy5 (1 eq., 50 µL, 1 mM in water) was combined with BCN-PEG6-PFP (20 eq., 50 µL, 20 mM in DMSO) and NaHCO_3_ (100 eq., 5 µL, 1 M in water). The mixture was incubated at 25 °C overnight under argon atmosphere and then the product was precipitated by acetone twice and purified by HPLC. After lyophilization, the conjugate was dissolved in water and concentration was measured using** a NanoDrop spectrophotometer (the total **yield = 67%).** The purity of the BCN-ON-Cy5 was evaluated by HPLC (Figure S4).

*
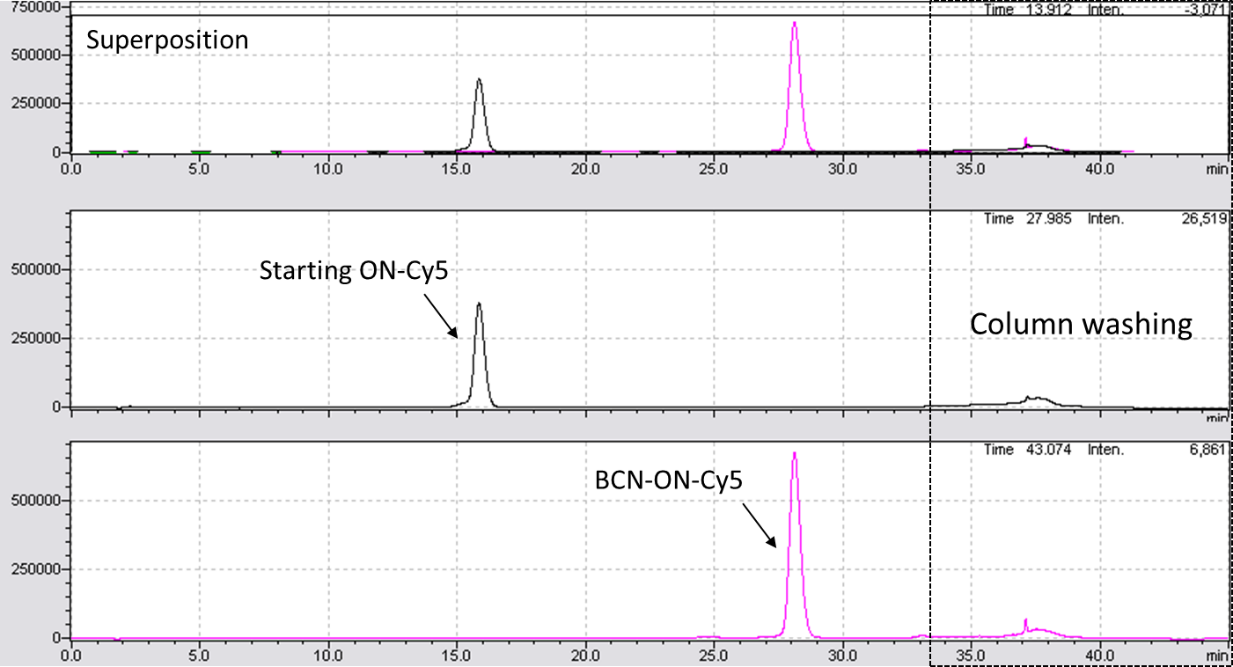
*

Figure S4. HPLC profile (detection at 260 nm, 15-35% B in 30 min) of the purified BCN-ON-Cy5.

### **Preparation of cON-MMAE**

**Thiol-modified oligonucleotide cON 5S (1 eq., 50 µL, 1 mM in water) was combined with PBS 10x** (10 µL), EDTA (1 µL, 0.5 M in water) **and TCEP (100 eq., 50 µL, 0.1 M in water) in a 2 mL Eppendorf tube. The mixture was incubated at 37 °C for 1 h and then the product was precipitated by acetone twice. The reduced oligonucleotide was dissolved in 100 µL of PBS 1x (pH 7.5, 5 mM EDTA) and then DMSO (94 µL) and APN-VC-MMAE (1.2 eq., 6 µL, 10 mM in DMSO) were added. The reaction mixture was incubated for 16 h at 25 °C and then the product was precipitated by acetone twice and was purified by HPLC. After lyophilization, the conjugate was dissolved in water and concentration was measured using** a NanoDrop spectrophotometer **(the total yield = 76%).** The purity of the cON-MMAE was evaluated by HPLC (Figure S5).


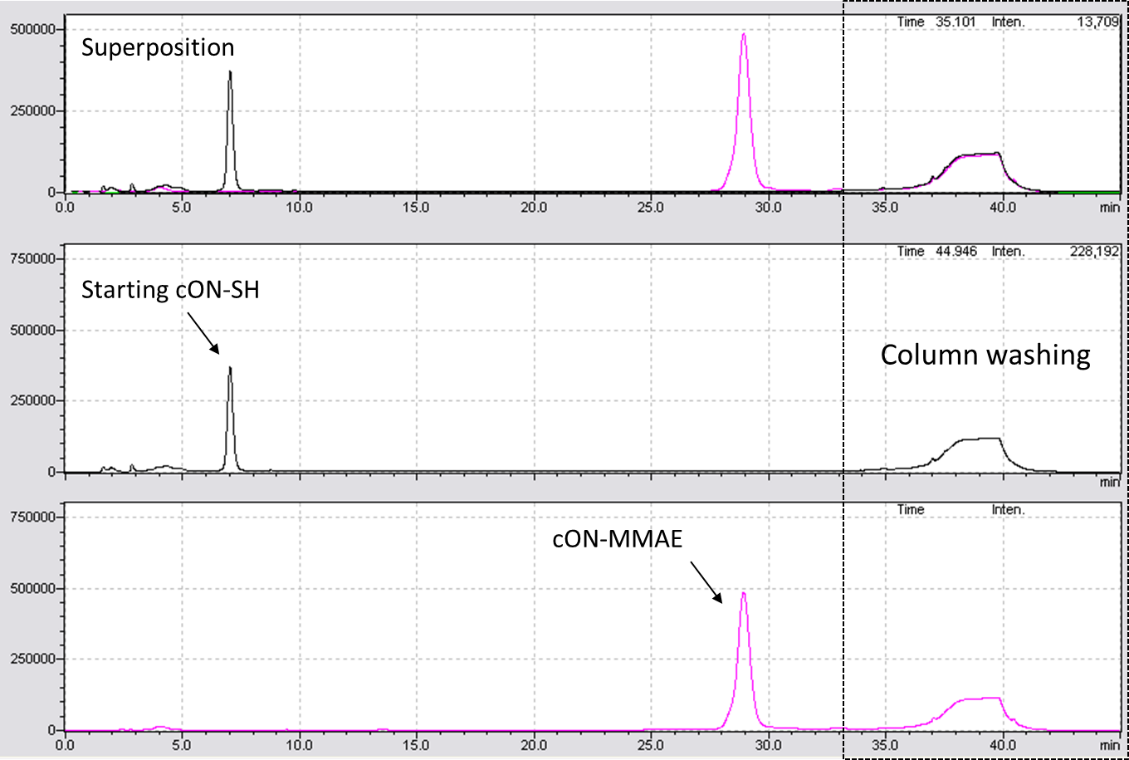


Figure S5. HPLC profile (detection at 260 nm, 10-40% B in 30 min) of the purified cON-MMAE.

## ****Preparation of antibody conjugates****

### Conjugate T-N_3_

4-azidobenzoyl fluoride (ABF) was synthetized as previously described.^3^ ABF (3 eq., 10 mM in DMSO, 2.06 µL) was added to a solution of trastuzumab (1 eq., 5 mg/mL, 34 µM, 200 µL in PBS 1x, pH 7.5) at room temperature and the reaction mixture was incubated at 25 °C for 30 min. The excess of reagents was then removed by gel filtration chromatography using Bio-spin P-30 Columns (Bio-Rad, Hercules, U.S.A.) pre-equilibrated with PBS (1x, pH 7.5) to give a solution of trastuzumab-azide conjugates **T-N_3_** in 98% yield.

### Conjugate T-ON-Cy5

**BCN-ON-Cy5** (2.9 eq., 229 µM in H_2_O, 55 µL) and 10 µL of PBS 10x was added to a solution of the **T-N_3_** conjugate (1 eq., 5.0 mg/mL, 34.25 µM, 125 µL in PBS 1x, pH 7.5). The mixture was purged with argon and incubated for 20 h at 25 °C. The conjugate was purified by gel filtration chromatography using AKTA Pure System (Superdex 200 Increase 10/300 GL, isocratic elution with PBS 1x, pH 7.5, 0.3 mL/min) to give **T-ON-Cy5** in 50% yield, which was characterized by SDS PAGE analysis, UV-Vis spectroscopy (Figure 2 and Table S1) and native SEC MS analysis (Figure S6). The same protocol was applied for the preparation of **T-ON** conjugate (without Cy5 on 3-end) using a 37-mer ON **NH2-ON**.

Figure S6. MS analysis of **T-ON-Cy5**: **(**A) Full mass spectrum, (B) Mass spectrum, (C) Deconvoluted mass spectrum, (D) DoC distribution chart.

### Conjugate T-DNA-Cy3

Conjugate T-DNA-Cy3 was prepared by mixing cON-Cy3 (3 eq.) with T-ON (1 eq.) in PBS 1x, pH 7.5.

### Conjugate T-DNA-MMAE

**BCN-ON-Cy5** (2.9 eq., 229 µM in H_2_O, 27.5 µL) and 5 µL of PBS 10x was added to a solution of the **T-N_3_** conjugate (1 eq., 5.0 mg/mL, 34.25 µM, 62.5 µL in PBS 1x, pH 7.5). The mixture was purged with argon and incubated for 20 h at 25 °C. The cON-MMAE (3.3 eq., 277 µM in H_2_O, 25.5 µL) was then added and the mixture was incubated for 30 min at 25 °C. The conjugate was purified by gel filtration chromatography AKTA Pure System (Superdex 200 Increase 10/300 GL, isocratic elution with PBS 1x, pH 7.5, 0.3 mL/min) to give **T-DNA-MMAE** in 58% yield, which was characterized by SDS PAGE 4-15% analysis, UV-Vis spectroscopy (Figure 2 and Table S1) and native SEC MS analysis (Figure S7).

Figure S7. MS analysis of **T-DNA-MMAE**: **(**A) SEC profile, (B) Mass spectrum, (C) Deconvoluted mass spectrum, (D) DAR distribution chart, (E) Full-length fluorescent gel of T-DNA-MMAE and DNA-Cy5 (positive payload control) showing no sign of unconjugated payload in the AOCs.

| Sample Name | 260 nm | 650 nm | C_BCA_(Ab), mg/mL | C(Ab), µM | C(Cy5), µM | DoC |
| --- | --- | --- | --- | --- | --- | --- |
| T-ON-Cy5 | 3.7 | 2.06 | 0.57 | 3.9 | 8.24 | 2.14 |
| T-DNA-MMAE | 7.1 | 2.48 | 0.68 | 4.6 | 9.92 | 2.16 |

Table S1. The average DoC values of AOCs calculated from UV-Vis spectra.

### Conjugate T-MMAE

DMSO (20 µL) was added to a solution of the **T-N_3_** conjugate (1 eq., 1.75 mg/mL, 150 µL in PBS 1x, pH 7.5), vortex and left for 2 min at rt. **BCN-VC-MMAE** (4 eq., 0.7 mM in DMSO, 10.1 µL) was then added. The final content of DMSO in the mixture was 17% v/v. The mixture was purged with argon and incubated for 20 h at 25 °C. The conjugate was purified by gel filtration chromatography using AKTA Pure System (Superdex 200 Increase 10/300 GL, isocratic elution with PBS 1x, pH 7.5, 0.3 mL/min) to give **T-MMAE** in 32% yield, which was characterized by native MS analysis (Figure S8).

Figure S8. MS analysis of **T-MMAE**: **(**A) Full mass spectrum, (B) Mass spectrum, (C) Deconvoluted mass spectrum, (D) DAR distribution chart

### Conjugate T-Cys-MMAE

Trastuzumab (1 eq., 5 mg/mL, 5 mL, 169 nmol) in PBS 1x (pH 7.5, 5 mM EDTA) was reduced with TCEP (2.2 eq., 0.1 M, 3.76 µL) for 2 h at 37 °C. Then **APN-VC-MMAE** (15 eq., 16.6 mM in DMSO, 155 µL, 2.57 µmol) was added and the reaction mixture was incubated overnight at 25 °C. The excess of payload was removed by stirring the reaction mixture with 100 mg of thiol beads (TentaGel S SH, IRIS Biotech, prewashed prior utilization with 3x DCM, 3x MeOH, 2x H2O, 3x PBS 1x) overnight at 25 °C. The mixture was then centriguged at 1000 g for 5 min and the supernatant was purified using AKTA Pure System (Superdex 200 Increase 10/300 GL, isocratic elution with PBS 1x, pH 7.5, 0.3 mL/min). The resulting conjugate **T-Cys-MMAE** was obtained in 72 % yield and was characterized by native MS analysis (Figure S9).

Figure S9. MS analysis of **T-Cys-MMAE**: **(**A) Full mass spectrum, (B) Mass spectrum, (C) Deconvoluted mass spectrum, (D) DAR distribution chart.

### **T-Fluor and T-ON-Fluor conjugates**

Trastuzumab or **T-ON** conjugate (average DoC of ~2) was concentrated to 1-5 mg/mL on micro-concentrators (Vivaspin, 50 kD cutoff, Sartorius, Gottingen, Germany) and reacted with 20 equiv. of FITC (10 mM in DMSO). The mixture was incubated at 25 °C overnight. The excess of FITC was then removed by gel filtration chromatography using Bio-spin P-30 Columns (Bio-Rad, Hercules, U.S.A.) pre-equilibrated with PBS 1x, pH 7.5 to give a solution of FITC-labelled antibody **T-Fluor** or AOC **T-ON-Fluor** with DoC_UV_(fluorescein) of 2 and 5, respectively.

Figure S10**.** Evaluation of conjugates stability in human plasma at 37 °C using in-gel fluorescence (Cy5 excitation) after SDS PAGE (4-15%). The bands of bromophenol blue correspond to a migration front of gel electrophoresis.

Figure S11. Binding kinetics assay for fluorescein labelled mAb **T-Fluor**, AOC **T-ON-Fluor** and free oligonucleotide **cON-Fluor** using LigandTracer assay.

Figure S12. (A) Denaturing SDS PAGE (4-15%) analysis showed complete hybridization of **T-DNA-MMAE** and no sign of unconjugated payloads (bands of **DNA-MMAE** and **ON-Cy5** served as a positive control). (B) Structure of the conjugates.

# References

(1) Dommerholt, J.; Schmidt, S.; Temming, R.; Hendriks, L. J. A.; Rutjes, F. P. J. T.; van Hest, J. C. M.; Lefeber, D. J.; Friedl, P.; van Delft, F. L. Readily Accessible Bicyclononynes for Bioorthogonal Labeling and Three-Dimensional Imaging of Living Cells. *Angew. Chemie* **2010**, *122* (49), 9612–9615.

(2) Kumar, A.; Erasquin, U. J.; Qin, G.; Li, K.; Cai, C. “Clickable”, Polymerized Liposomes as a Versatile and Stable Platform for Rapid Optimization of Their Peripheral Compositions. *Chem. Commun.* **2010**, *46* (31), 5746.

(3) Dovgan, I.; Ursuegui, S.; Erb, S.; Michel, C.; Kolodych, S.; Cianférani, S.; Wagner, A. Acyl Fluorides: Fast, Efficient, and Versatile Lysine-Based Protein Conjugation via Plug-and-Play Strategy. *Bioconjug. Chem.* **2017**, *28* (5), 1452–1457.
